# The Salford Nature Environments Database (SNED): an open-access database of standardized high-quality pictures from natural environments

**DOI:** 10.3758/s13428-024-02556-4

**Published:** 2024-12-19

**Authors:** Robert C. A. Bendall, Sam Royle, James Dodds, Hugh Watmough, Jamie C. Gillman, David Beevers, Simon Cassidy, Ben Short, Paige Metcalfe, Michael J. Lomas, Draco Graham-Kevan, Samantha E. A. Gregory

**Affiliations:** 1 School of Health and Society, University of Salford, Allerton Building, Frederick Road, Salford, M5 4WT UK; 2 Centre for Applied Health Research, University of Salford, Salford, UK

**Keywords:** Salford Nature Environments Database, SNED, Natural environments, Cognitive restoration, Mental health, Wellbeing, Environmental psychology, Attention restoration theory, Nature

## Abstract

The growing interest in harnessing natural environments to enhance mental health, including cognitive functioning and mood, has yielded encouraging results in initial studies. Given that images of nature have demonstrated similar benefits, they are frequently employed as proxies for real-world environments. To ensure precision and control, researchers often manipulate images of natural environments. The effectiveness of this approach relies on standardization of imagery, and therefore, inconsistency in methods and stimuli has limited the synthesis of research findings in the area. Responding to these limitations, the current paper introduces the Salford Nature Environments Database (SNED), a standardized database of natural images created to support ongoing research into the benefits of nature exposure. The SNED currently exists as the most comprehensive nature image database available, comprising 500 high-quality, standardized photographs capturing a variety of possible natural environments across the seasons. It also includes normative scores for user-rated (801 participants) characteristics of fascination, refuge and prospect, compatibility, preference, valence, arousal, and approach–avoidance, as well as data on physical properties of the images, specifically luminance, contrast, entropy, CIELAB colour space parameter values, and fractal dimensions. All image ratings and content detail, along with participant details, are freely available online. Researchers are encouraged to use this open-access database in accordance with the specific aims and design of their study. The SNED represents a valuable resource for continued research in areas such as nature-based therapy, social prescribing, and experimental approaches investigating underlying mechanisms that help explain how natural environments improve mental health and wellbeing.

## Introduction

Our environment is a key determinant of mental health (Clark et al., 2007), with research in the field providing convincing evidence demonstrating the benefits of nature exposure for outcomes such as reduced psychological distress (Astell-Burt et al., 2013), improved mood (Roberts et al., 2019), and overall wellbeing (O’Brien & Forster, 2021). Enhanced cognitive functioning is also evident, including improved selective and sustained attention (Vella-Brodrick & Gilowska, 2022), as well as enhanced working and short-term memory (Lega et al., 2021). Interest in the benefits of nature exposure has increased in recent years due to the COVID pandemic, with this period highlighting the benefits of access to nature and green spaces as important aspects in promoting wellbeing and mental health (O’Brien & Forster, 2021).

A key focus for researchers is investigating the restorative properties of nature on health, including cognitive wellbeing. Among restoration research, a consistent finding is the advantages of natural space over urban settings, with research showing benefits of green infrastructure in leisure spaces (Fu & Xue, 2023), educational settings (Vella-Brodrick & Gilowska, 2022), and hospitals (Donovan et al., 2019; Ulrich, 1984). Studies show that even a 40-s exposure to a green environment can have positive cognitive effects (Lee et al., 2015) and, critically, that images and videos of nature are also restorative (Berman et al., 2008; Berto, 2005; Mayer et al., 2009; Pilotti et al., 2015).

While we know that natural environments are capable of conjuring such beneficial effects, the underlying mechanisms of why we find nature so restorative remain the subject of debate among researchers. The dominant theories in the area of nature-based restoration include stress reduction theory (Ulrich, 1983; Ulrich et al., 1991), attention restoration theory (Kaplan, 1995), and the perceptual fluency account (Joye et al., 2016; Joye & van den Berg, 2011). Stress reduction theory posits that we find nature restorative because we have an evolutionary bias towards natural environments where we would find food and shelter. As a result, unthreatening natural settings lead to a reduction in stress-related muscle tension and heart rate (Ulrich et al., 1991). Closely related is prospect–refuge theory (Appleton, 1984), whereby we favour environments that provide a place of shelter or safety from which to explore (see also Kaplan et al., 1989). Attention restoration theory proposes a more mechanistic theoretical approach, arguing that natural environments can restore attentional capacities—thereby aiding cognition—by allowing individuals to be removed from everyday stressors, to experience the scope of possibility in the environment (i.e., extent), to be in environments compatible with their basic motivations, and to experience a softly fascinating visual environment (Kaplan, 1995, but see Joye & Dewitte, 2018, for a critique). This theory provides the foundation for the perceived restorativeness scale of natural environments (Hartig et al., 1997; Korpela & Hartig, 1996), a measure of an individual’s perceptions of the restorative nature of the environments that they spend time in. The perceptual fluency account involves soft fascination, a concept referred to in attention restoration theory. Soft fascination is the notion that natural environments are able to hold our attention in a way that is effortless, restoring our attentional capacity (Kaplan, 1995). The perceptual fluency account specifically relates to the idea that the repetitive patterns in natural environments such as leaves in trees make them softly fascinating and consequently easier to process (Franěk et al., 2019; Joye et al., 2016). An alternative account of restoration has recently been proposed whereby nature is cognitively restorative because it satisfies a hedonic goal, where a needed and desired break from tasks is afforded (Joye et al., 2022).

Such theories offer a basis from which to gain an understanding of the positive effects of nature. However, questions remain as to whether some natural environments are more restorative than others (Wyles et al., 2019). Research suggests that specific properties of environments can make them more, or less, restorative. For instance, higher fractal dimensions (i.e., more repeating patterns) in nature scenes have been associated with cognitive restoration via increased perceptual fluency (Joye et al., 2016; Joye & van den Berg, 2011), and changes in eye movements (Franěk et al., 2019). Further, research indicates that the colour properties of an environment can affect preference for an environment, as well as mood and wellbeing (for a review see Jalil et al., 2012). Additionally, individual differences can also affect the restorative properties of an environment. Research suggests that we respond to environments with an immediate judgement of whether we like or dislike them (Zajonc, 1980), and while it is likely that the restorative nature of an environment may affect this judgement, it is also possible that our preference could affect how restorative we find the environment. For example, researchers have shown that environmental preferences affect how restorative people expect an environment to be (Wilkie & Stavridou, 2013). We therefore need to better understand both the individual differences in how people perceive a wide variety of apparently restorative environments, and the variations in the properties of those environments. Critical for researchers investigating environmental influences on cognitive and affective processes is the availability of standardized stimuli. A current lack of consistency across types of stimuli and methods used in environmental restoration research makes assimilating findings from these studies into a coherent account of the phenomenon problematic. Past research has predominantly focused on whether nature environments are more restorative than urban settings, and the amount of exposure to green space required for evidence of restoration to be shown (for meta-analysis, see Menardo et al., 2021). More recently, the focus has shifted towards the restorative qualities of different types of nature whilst attempting to identify the qualities of nature most likely to trigger psychological restoration (e.g., Wyles et al., 2019). All types of nature, from wilderness (Milligan et al., 2021) to urban parks (Weber & Trojan, 2018), to back gardens (Howarth et al., 2020; Van Den Berg & Custers, 2011) and even allotment gardening (Wood et al., 2016), have been shown to be restorative. Embarking on a fine-grained analysis of the individual characteristics of these contrasting nature types is a logical and crucial next step for research in the area.

To the best of our knowledge, there is only one standardized database dedicated to images of natural environments.^1^ The e-Nature Positive Emotions Photography Database (e-NatPOEM; Dal Fabbro et al., 2021) comprises 433 images of positive natural environments. Images in the e-NatPOEM are rated for valence, arousal, and a single word describing the evoked feeling. Whilst the database provides a useful resource for researchers, it does not provide standardized ratings of potentially relevant aspects of the photographed environments or detailed demographic information on the image raters, such as cultural factors, that may need to be considered. It is also often critical for researchers to have information regarding the physical properties of stimuli used in research, such as luminance and colour information, as such properties have been shown to influence visual attention (e.g., Bradley et al., 2007; Codispoti & De Cesarei, 2007; Franěk et al., 2019). These properties are not currently available as part of the e-NatPOEM; however, this information is available in image databases such as the Nencki Affective Picture System (NAPS; Marchewka et al., 2014).

### The Salford Nature Environments Database (SNED)

Given the increased focus on nature-based therapy and social prescribing, in conjunction with experimental approaches investigating the mechanisms linking natural environments with improved mental health and wellbeing, there is a need for a standardized picture database comprising different natural environments. In the current work, we provide a novel database of 500 high-quality, standardized photographs capturing a variety of possible natural environments across the seasons: water, woodlands, mountains, deserts, fields, snow, caves, managed landscapes, and urban environments. Predicated on theoretical restoration research, normative ratings are provided separately for mystery and interest (capturing the characteristic of fascination; as per Hartig et al., 1997), feeling at ease (capturing the concept of prosect and refuge; Appleton, 1984), familiarity (capturing the concept of compatibility; Hartig et al., 1997), and pleasantness (capturing preference factors; Zajonc, 1980). Using the dimensional category theory of emotion (for a review see Mauss & Robinson, 2009), and in accordance with previous databases (e.g., NAPS, Marchewka et al., 2014; e-NatPOEM, Dal Fabbro et al., 2021), measures of valence, arousal, and approach–avoidance were also recorded. Further, in line with the NAPS database (Marchewka et al., 2014), we provide information on physical properties of the images, specifically luminance, contrast (global), entropy (first order), CIELAB colour space parameter values, and—novel here—fractal dimension. Additionally, to permit consideration of inter-individual and cultural differences between image raters, we provide participant demographic information related to age, gender identity, ethnicity, geographical location, education background, economic status, and environmental preference.

Urban images included in the database are primarily intended to serve as control stimuli such that properties of the nature stimuli can be compared to supposed non-restorative stimuli. Hence, for the purpose of validation analyses comparing nature images with urban (control) images, we made the following directional and preregistered hypotheses; we predicted that compared to urban images, nature images would be rated higher in positive valence, less arousing, greater in approach tendencies, and more pleasant. Additional non-directional and preregistered (https://osf.io/sbqg8) hypotheses predicted that nature images would differ from urban images in levels of mystery, interest, familiarity, and prospect/refuge. The Salford Nature Environments Database (SNED) was created to help advance rigour and consistency in nature exposure research and is therefore made freely available to the scientific community for non-commercial use. The open-access database is available on the Open Science Framework (https://osf.io/qm42t/) and is available in the KAPODI searchable database of emotional stimuli sets (Diconne et al., 2022).

## Method

### Participants

We recruited 801 participants (394 female, 391 male, 10 non-binary, 6 preferred not to say) aged 18–79 years (*M* = 32.0, *SD* = 10.6). To improve the validity and generalizability of our research, we attempted to source participants from a diverse population, attempting to recruit outside of traditional participant pools that contain people from WEIRD (Western, educated, industrialized, rich, and democratic) backgrounds. Therefore, using the Prolific platform (www.prolific.co), we recruited English-speaking participants from eight geographical regions: Africa, Arab States and Middle East, Asia and Pacific (excluding Australia and New Zealand), Australia and New Zealand, Europe (excluding the UK), United Kingdom, North America, and South and Latin America. Our initial recruitment target was 800 participants distributed across these regions based on size of region and availability of participants on Prolific, meaning recruitment was skewed towards the UK, Europe, and North America. For recruitment targets and actual recruitment by region (as well as countries included in these regions) please see https://osf.io/qm42t/. Participants were paid £8 for taking part. For a further breakdown of participant demographics see Table 1.

**Table 1:** Participant demographics

	All *N* (%)	Africa	Arab States and Middle East	Asia and South Pacific^a^	Australia and New Zealand	Europe^b^	United Kingdom	North America	South and Central America
Number of participants	801	100	51	150	50	151	99	98	102
Gender									
Male	391 (48.81)	47 (47.00)	23 (45.10)	77 (51.33)	23 (46.00)	76 (50.33)	47 (47.48)	50 (51.02)	48 (47.06)
Female	394 (49.19)	52 (52.00)	26 (50.98)	71 47.33)	23 (46.00)	70 (46.36)	52 (52.52)	47 (47.96)	53 (51.97)
Non-binary	10 (1.25)	0	1 (1.96)	0	3 (6.00)	4 (2.65)	0	1 (1.02)	1 (0.98)
Prefer not to say	5 (0.62)	1 (1.00)	1 (1.96)	2 (1.33)	1 (2.00)	0	0	0	0
Other	1 (0.13)	0	0	0	0	1 (0.66)	0	0	0
Age (years)									
Range	18–79	20–53	20–65	19–61	20–59	18–67	20–73	21–79	20–64
Mean (standard deviation)	32.00 (10.57)	27.56 (6.83)	31.88 (10.11)	31.03 (8.84)	33.58 (9.44)	27.62 (7.53)	37.56 (10.96)	40.19 (14.64)	30.30 (8.61)
Education									
No secondary/high school education	2 (0.25)	0	1 (1.96)	0	0	0	0	1 (1.02)	0
University/college degree	355 (44.32)	50 (50.00)	17 (33.33)	67 (44.67)	31 (62.00)	49 (32.45)	43 (43.43)	41 (41.84)	57 (55.88)
Postgraduate degree	164 (20.47)	11 (11.00)	19 (37.26)	54 (36.00)	5 (10.00)	31 (20.53)	16 (16.16)	15 (15.31)	13 (12.75)
Secondary school/exams completed	87 (10.86)	13 (13.00)	7 (13.73)	6 (4.00)	3 (6.00)	24 (15.90)	18 (18.18)	10 (10.20)	6 (5.88)
Some secondary school, no exams	17 (2.12)	0	1 (1.96)	3 (2.00)	2 (4.00)	5 (3.31)	1 (1.01)	3 (3.06)	2 (1.96)
Some university/college	143 (17.85)	22 (22.00)	4 (7.84)	18 (12.00)	4 (8.00)	37 (24.50)	13 (13.13)	23 (23.47)	22 (21.57)
Trade/technical/vocational training	33 (4.12)	4 (4.00)	2 (3.92)	2 (1.33)	5 (10.00)	5 (3.31)	8 (8.08)	5 (5.10)	2 (1.96)
MacArthur scale score									
Mean (standard deviation)	5.58 (1.55)	5.49 (1.56)	5.88 (1.77)	6.04 (1.50)	5.50 (1.52)	5.56 (1.45)	5.11 (1.53)	5.26 (1.64)	5.65 (1.44)
Reports as top third (8th – 10th rung)	69 (8.61)	6 (6.00)	14 (27.45)	22 (14.67)	4 (8.00)	9 (5.96)	3 (3.03)	4 (4.08)	7 (6.87)
Reports as middle (4th – 7th rung)	643 (80.28)	82 (82.00)	32 (62.75)	118 (78.66)	42 (84.00)	127 (84.11)	78 (78.79)	78 (79.59)	86 (84.31)
Reports as bottom third (1st – 3rd rung)	89 (11.11)	12 (12.00)	5 (9.80)	10 (6.67)	4 (8.00)	15 (9.93)	18 (18.18)	16 (16.33)	9 (8.82)
Rural vs urban scales									
Living location rural (responded ≤ 5)	138 (17.23)	12 (12.00)	4 (7.84)	17 (11.33)	3 (6.00)	34 (22.52)	35 (35.35)	29 (29.59)	4 (3.92)
Living location urban (responded ≥ 6)	662 (82.77)	88 (88.00)	47 (92.16)	133 (88.67)	47 (94.00)	117 (77.48)	64 (64.65)	69 (70.41)	98 (96.08)
Mean (standard deviation)	7.50 (2.23)	7.93 (2.18)	8.43 (1.85)	7.88 (1.70)	7.52 (1.67)	7.18 (2.32)	6.28 (2.52)	6.80 (2.49)	8.36 (1.80)
Current location rural (responded ≤ 5)	128 (15.98)	8 (8.00)	4 (7.84)	17 (11.33)	3 (6.00)	30 (19.87)	30 (30.30)	31 (31.63)	5 (4.90)
Current location urban (responded ≥ 6)	673 (84.02)	92 (92.00)	47 (92.16)	133 (88.67)	47 (94.00)	121 (80.13)	69 (69.70)	67 (68.37)	97 (95.10)
Mean (standard deviation)	7.60 (2.18)	8.28 (1.83)	8.41 (1.94)	7.91 (1.84)	7.52 (1.69)	7.41 (2.28)	6.50 (2.44)	6.69 (2.46)	8.30 (1.82)
Most time spent in rural location (responded ≤ 5)	127 (15.86)	10 (10.00)	4 (7.84)	16 (10.67)	3 (6.00)	25 (16.56)	32 (32.32)	29 (29.59)	8 (7.84)
Most time spent in urban location (responded ≥ 6)	674 (84.14)	90 (90.00)	47 (92.16)	134 (89.33)	47 (94.00)	126 (83.44)	67 (67.68)	69 (70.41)	94 (92.16)
Mean (standard deviation)	7.62 (2.12)	8.10 (1.89)	8.37 (1.84)	8.02 (1.74)	7.62 (1.63)	7.58 (2.03)	6.24 (2.40)	6.67 (2.42)	8.34 (1.93)
Disliked rural leisure time (responded ≤ 5)	223 (27.84)	47 (47.00)	14 (27.45)	37 (24.67)	19 (38.00)	30 (19.87)	14 (14.14)	29 (29.59)	33 (32.35)
Liked rural leisure time (responded ≥ 6)	578 (72.16)	53 (53.00)	37 (72.55)	113 (75.33)	31 (62.00)	121 (80.13)	85 (85.86)	69 (70.41)	69 (67.65)
Mean (standard deviation)	6.80 (2.40)	5.67 (2.84)	7.10 (2.27)	6.87 (2.28)	6.18 (2.30)	7.38 (2.03)	7.62 (2.00)	6.55 (2.53)	6.57 (2.46)
Disliked urban leisure time (responded ≤ 5)	248 (30.96)	23 (23.00)	17 (33.33)	40 (26.67)	7 (14.00)	52 (34.44)	48 (48.49)	39 (39.80)	22 (21.57)
Liked urban leisure time (responded ≥ 6)	553 (69.04)	77 (77.00)	34 (66.67)	110 (73.33)	43 (86.00)	99 (65.56)	51 (51.51)	59 (60.20)	80 (78.43)
Mean (standard deviation)	6.43 (2.43)	7.34 (2.46)	6.49 (2.32)	6.83 (2.42)	7.30 (1.83)	6.49 (2.40)	5.46 (2.38)	6.14 (2.49)	7.28 (2.18)

^a^ Excludes Australia and New Zealand

^b^ Excludes UK

Our sample size was based on previous work reporting the creation of similar pictorial databases (NAPS: Marchewka et al., 2014; e-NatPOEM: Dal Fabbro et al., 2021). Pictures in the NAPS were rated on average 55 times. The authors of the e-NatPOEM report a sample size calculation suggesting a minimum sample of 41 ratings is required, and in their study, participants rated each picture between 36 and 108 times (*Mdn* = 57). In the current work, each property within each picture was rated between 154 and 168 times (*M* = 160).

Ethical approval was obtained from the School of Health and Society Ethics Committee at the University of Salford prior to data collection. Stimuli were presented using Gorilla (www.Gorilla.sc; Anwyl-Irvine et al., 2020), an online study platform. Participants accessed the study through a web browser using their own desktop/laptop computers; participation via mobile phones or tablets was prohibited.

### Stimuli and materials

#### Images

The database contains 500 images, among which 435 are of natural scenes and 65 are control images showing urban scenes. The images were selected and processed as follows: images were collected by three research assistants using the online photo-sharing site Flickr, where images were filtered for public domain copyright status (CC0: meaning that the authors of the images reserved no rights to those images and so they could be used in databases such as the one reported here). Initial collection was based on searches for images that fit within six categories: images containing water, woodlands, landscapes, caves, managed landscapes (i.e., farmland, reservoirs, and parks), and control (i.e., urban). Searches in the landscapes category were split into four further subcategories—desert, mountain, field, and snow—to ensure a variety of environments. Control images were described as images having little to no greenery or natural elements, such as cityscapes, street settings, and tramlines. Images containing people, animals, easily recognizable areas, obvious editing, and poor lighting were avoided. We aimed to include images with a minimum resolution of 1600 × 1200, and most images met this threshold; however, due to some discrepancies around image shape, we retained two images with a height below 1200 in the final database. All images were listed as CC0 at the time of collection, and a reverse search was run using Google Images to check that the image was from its original owner and there were no obvious copyright disputes related to the image. Additionally, at the time of collection, image address, image size, date added, date taken, and exchangeable image file format (EXIF) information availability were also recorded. At this initial stage, 845 images were collected.

Images were then curated over three phases. Within the initial phase, all images were checked for visual quality and against the search criteria. Images were excluded if they were obviously of low quality and did not fit the search criteria. This resulted in 601 images: 127 water images, 128 woodlands images, 173 landscape images, 80 managed landscape images, 20 cave images, and 73 control images. Images were then checked for duplicates, resulting in a total image set of 591. For final eliminations, images were reassessed using the search criteria with additional screening for image shape to ensure quality after being cropped and resized. Care was taken to ensure a range of images were retained, including being representative of seasonal changes. We therefore retained 500 images (65 urban control, 435 nature), representative of the initial search categories: 108 water images, 114 woodlands images, 126 landscape images (31 desert, 28 field, 35 mountains, 32 snow), 70 managed landscape images, 17 cave images, and 65 control images.

For image presentation, images were cropped using the PhotoScape program (www.photoscape.org) to a standardized 16 × 19 ratio, suitable for presentation on standard computer monitors. This involved one researcher manually loading the images and cropping them to ensure the main contents of the images were preserved. For online image presentation purposes, images were resized (again using PhotoScape) to a resolution of 1920 × 1080, preserving the aspect ratio. Note that we had 10 images where the initial width was below 1920 (min 1600). For these images, the PhotoScape program used the bicubic interpolation method to increase the size of the image. Images in both their original form and resized form can be downloaded, but note that image statistics and ratings were performed on these resized versions.

#### Rating scales

 Participants rated the images on the following properties using a bipolar semantic sliding scale based on similar studies (i.e., e-NatPOEM: Dal Fabbro et al., 2021; NAPS: Marchewka et al., 2014; Geneva Affective Picture Database: Dan-Glauser & Scherer, 2011). Arousal: “Looking at this picture makes me feel …” (from 1 = relaxed to 9 = aroused). Valence: “I perceive this picture as…” (1 = very negative to 9 = very positive). Approach/avoidance: “My reaction to this picture is…” (from 1 = to avoid 9 = to approach). Pleasantness: “I find looking at this picture…” (from 1 = very unpleasant to 9 = very pleasant). Participants also rated the environment in the images based on properties related to restoration theories. Familiarity: “The environment in this picture feels…” (from 1 = very unfamiliar to 9 = very familiar). Mystery: “If I was to further explore the environment in this picture, I think there would be…” (from 1 = little to discover to 9 = lots to discover). Interest: “I find the environment in this picture…” (from 1 = not interesting to 9 = very interesting). Prospect/refuge: “I think the environment in this picture is somewhere I would feel …” (from 1 = uneasy to 9 = at ease). Ratings were always presented in the same order to avoid participant errors. To check that participants were not clicking randomly without reading, we also included three awareness checks asking the participants to answer yes or no to whether a presented image contained a car. No participant met the preregistered threshold of failing two of these checks.

Social class was measured using the MacArthur scale (Adler et al., 2000). Here, participants were shown a ladder with 10 rungs and were told, “The ladder represents where people stand in society. At the top of the ladder are the people who are the best off, those who have the most money, most education, and best jobs. At the bottom are the people who are the worst off, those who have the least money, least education, worst jobs, or no job. Please indicate where you think you stand on the ladder.” We chose to use this scale due to the cross-cultural element of the study, making income-related measures somewhat meaningless.

We also used a rural to urban scale (Cox et al., 2018) to record the types of environments the participant spent time in, and how much they enjoyed spending time in these types of environments. The rural to urban scale asked participants to rate on a scale of 1 to 10—where 1 is an isolated place in the country, 6 is in the suburbs, and 10 is in the middle of a city—where they lived, their current location, and where they spent most of their time. We then also asked how much they liked to spend their leisure time in an urban environment and how much they liked to spend their leisure time in a rural environment on a scale of 1 to 10, where 1 is “do not like” to 10 “like very much”.

#### Physical properties of images

The physical properties of each image were calculated using a Python script using NumPy, SciKit Image, and Pillow libraries (see https://osf.io/qm42t/ for the Python script used). To calculate luminance (i.e., the “lightness” of an image absent any chromatic information), we first converted the images to greyscale and then calculated the average pixel value of the greyscale image. Contrast (i.e., the variation between the light and dark parts of the image) was calculated as the standard deviation across all pixels of the greyscale image (Bex & Makous, 2002). Entropy (i.e., the randomness/complexity of an image) was calculated using Shannon’s (1948) method. CIELAB colour space values were calculated as an average for each channel for each image (Poynton, 2015). CIELAB values are based on the opponent-process theory of colour vision, providing a luminance (L*) value, as well as two chromatic measures. The a* channel provides information on a scale from green (negative values) to red (positive values), with the b* channel providing information on a scale from blue (negative values) to yellow (positive values). This colour space is thought to more closely approximate aspects of human vision than the RGB (red, green, and blue) colour space (Tkalcic & Tasic, 2003). These values reflect those provided in the NAPS (Marchewka et al., 2014) and give basic information about how much blue or green is in the images, as these are crucial restorative properties in nature images (e.g., White et al., 2020). The fractal dimension (i.e., a measure of the self-similarity of the image) was calculated using the box-counting method, which involves the coding of the image into a binary array representing “objects” and background, overlaying boxes of various pixel sizes over the image, and counting the number of boxes that contain part of the objects. The fractal dimension is thus calculated as the gradient of the slope relating the logarithm of the number of boxes (log_10_) containing object pixels and the logarithm of the size of the boxes (Burtan et al., 2021; Foroutan-pour et al., 1999). Values for these physical property measures are included with the image-level data in the project GitHub (https://github.com/Salford-PsyTech/Salford_Nature_Environments_Database).

### Design

Each participant rated 100 images on each of the eight properties. The 500 images were therefore grouped into 10 sets of 100 images via hard-coded pseudo-randomization to ensure an overlap between image sets, so that each image would be rated an equal number of times across the study. To achieve this, each image was pseudo-randomly assigned to both one of the first five image sets (sets 1–5), and one of the last five image sets (sets 6–10). Each image therefore appeared in two sets, with differing images being rated alongside. Each image set contained a range of images from across the databases associated with a variety of scene types. The presentation order of these images was pseudo-randomized using a custom script, with coded rules in place to reduce image type repetition (e.g., multiple rivers in a row). This was achieved by initiating a counter that increased if the category of the image randomly assigned to a trial was the same as that assigned to the previous trial. If the counter indicated that three images from that category would be shown in a row, then images from that category were excluded from the next image selection. In the case that the number of images in any one category still to be assigned to trials equalled or exceeded the number of images in all other categories, an approach was adopted wherein the selection of images alternated between the large category and then all other categories. A copy of the experiment, including the rating task (and randomization script), has been made available via Gorilla Open Materials (https://app.gorilla.sc/openmaterials/685394).

### Procedure

**Fig. 1:**
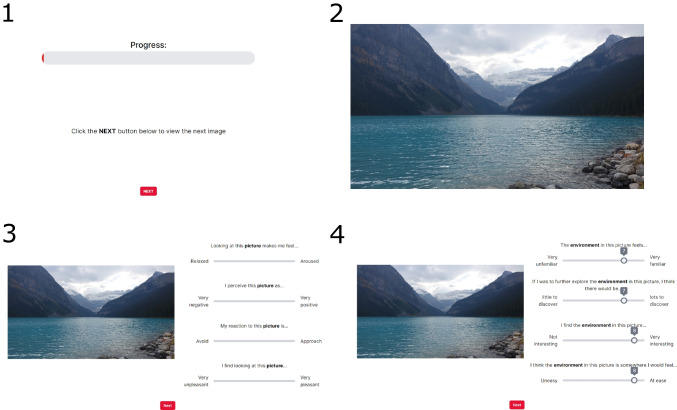
Trial procedure for image rating task. (**1**) Participants saw a progress bar between images and initiated each rating phase using the “next” button, allowing them to take breaks as needed. (**2**) The image was then displayed alone for 3 s. (**3**) A smaller version of the image was shown alongside the Likert scales asking about feelings towards the image in terms of arousal, valence, approach/avoid, and pleasantness. (**4**) Likert scales asking about feelings towards the environment in the picture in terms of familiarity, mystery, interest, and prospect/valence were next shown alongside the image. Slides were always shown in this order. All Likert scales had to be selected to allow participants to click “next” to progress; there was no time limit for this

Once informed consent had been obtained, participants provided demographic information (age, gender, ethnicity, nationality, place of birth, level of education, social class). Subsequently, participants were asked about the types of environments where they lived and spent most of their leisure time. Participants were then presented with the task instructions and the series of images for rating. Each image was presented in full-screen view first for 3 s before being minimized alongside the rating scales (see Fig. 1). Once ratings for the image had been completed, participants could move on to the next image by pressing next when ready to continue. Participation in the study took approximately 1 h.

## Results

Of 801 participants included in the sample, 790 had 800 data points, as expected. There were five participants with less than this number: one who only made 287 ratings due to issues completing the study and four who made 796 ratings, potentially due to computer error. In addition, we had five participants who had duplicate ratings; three had 804, one had 808, and one had 813. These extra ratings were due to computer error, as the ratings and time stamps were the same for the two ratings, and thus we removed the duplicates. The final database comprised 640,267 ratings from 801 participants (*M* = 160 ratings per parameter for each image, min 154, max 168). For each of the pictures presented, an average of all responses for each rating was calculated.

### Validation of rated properties

We compared aggregate data (i.e., using the ratings for each image as an average across participants) for valence, arousal, approach–avoidance, and pleasantness ratings for the images of nature (*n* = 435) in comparison to the urban control images (*n* = 65) using independent-samples *t*-tests. As we made directional hypotheses, uncorrected one-sided *p* values are reported. For all comparisons, Levene’s test for equality of variances was significant; therefore, we used Welch’s *t*-test in JASP 0.15 (JASP Team, 2021). As predicted, results confirmed that the images of nature were rated significantly differently to the urban images across all ratings. Participants rated the nature images as evoking higher positive valence (*M* = 6.657, *SD* = 0.824) than the urban control images (*M* = 5.137, *SD* = 0.592), *t*(105.175) = 18.227, *p* < 0.001, *d* = 2.118. Participants rated the nature images as evoking lower arousal (*M* = 3.737, *SD* = 0.775) than the urban control images (*M* = 5.230, *SD* = 0.365), *t*(167.824) = 25.485, *p* < 0.001, *d* = 2.465. Participants rated the nature images as evoking higher approach tendencies (*M* = 6.346, *SD* = 0.969) than the urban control images (*M* = 5.260, *SD* = 0.705), *t*(104.073) = 10.968, *p* < 0.001, *d* = 1.281. Finally, participants rated the nature images as evoking higher pleasantness (*M* = 6.621, *SD* = 0.893) than the urban control images (*M* = 5.079, *SD* = 0.659), *t*(102.743) = 16.695, *p* < 0.001,* d* = 1.964.

**Fig. 2:**
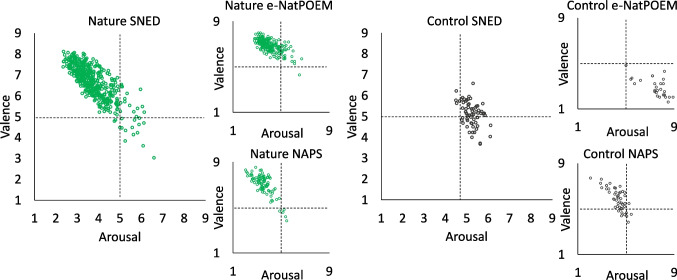
Affective space of the SNED image database for the nature and control images, with the mean aggregate ratings of valence and arousal for each image. The affective spaces of comparable images from the e-NatPOEM and NAPS databases are also presented

We also visually compared our ratings against those of similar databases, NAPS (Marchewka et al., 2014) and e-NatPOEM (Dal Fabbro et al., 2021); see Fig. 2. We selected nature images which corresponded to ours, thus using only the landscape images of natural environments from NAPS and excluding images where the focus was on animals for the e-NatPOEM. For the control images, we again selected only images from the landscapes category that had correspondence to ours from the NAPS (i.e., images of built environments). However, for the e-NatPOEM, we used all control images, as there were only 28 available; therefore, it is important to note that in addition to images of cities, these include images such as dead animals and destruction. Like these databases, our data show a linear relationship between arousal and valence. Further, like the e-NatPOEM and NAPS, our images of nature fall largely in the upper left quartile of affective space, with low arousal and high valence ratings. Our control images lie more centrally than those of the e-NatPOEM, which show high arousal and low valence.

In addition, we compared aggregate data (i.e., using the ratings for each image as an average across participants) for ratings on the other factors between the nature images and the control images in our study. These comparisons were planned but we made no directional hypotheses; therefore, the uncorrected two-sided test values are used. Here, for the interest and mystery ratings, Student’s *t*-test could be used, whereas for prospect/refuge and familiarity, equal variances could not be assumed, and thus Welch’s test was used. We found that participants overall rated the nature images as evoking a significantly lower feeling of familiarity (*M* = 4.910, *SD* = 0.966) than the urban control images (*M* = 6.102, *SD* = 0.447), *t*(172.324) = 16.504, *p* < 0.001, *d* = 1.584. This is likely related to the diversity of the natural images shown. Participants rated the nature images as significantly more interesting (*M* = 6.301, *SD* = 0.832) than the urban control images (*M* = 4.988, *SD* = 0.794), *t*(498) = 11.936, *p* < 0.001, *d* = 1.587. Participants also rated the nature images as having significantly more to discover (higher mystery) (*M* = 5.933, *SD* = 0.769) than the urban control images (*M* = 5.696, *SD* = 0.873), *t*(498) = 2.271, *p* = 0.024, *d* = 0.302. Finally, we found that the participants overall rated the nature images as being of somewhere they were more likely to feel at ease (prospect/refuge) (*M* = 6.135, *SD* = 1.028) compared to the urban control images (*M* = 5.206, *SD* = 0.605), *t*(127.562) = 10.346, *p* < 0.001, *d* = 1.101.

### Reliability of rated properties

To check the reliability of ratings in the image-level data, we used JASP to conduct the Spearman–Brown-corrected split-half method whereby the dataset is split into two separate subsets. We made sure that images from each image category were split equally (or as equally as possible when categories contained an odd number of items) across the two subsets by alternating which subset each image went into within each image category (i.e., urban control, caves, water, etc.). Spearman–Brown-corrected split-half estimates of rating reliability for the image-level data varied from 0.673 for mystery to 0.842 for arousal, therefore within an acceptable range of reliability. Full results are presented in Table 2.

**Table 2:** Spearman’s correlations between rated properties, fractal dimensionality, and the a* (green–magenta) and b* (blue–yellow) channels of the CIELAB parameters

Property	Split-half reliability^a^	1	2	3	4	5	6	7	8	9	10
1. Approach/avoid	0.724	–									
2. Arousal	0.842	− 0.869**	–								
3. Familiarity	0.799	0.196**	− 0.255**	–							
4. Interest	0.755	0.804**	− 0.567**	− 0.227**	–						
5. Mystery	0.673	0.581**	− 0.273**	− 0.078	0.804**	–					
6. Pleasantness	0.785	0.951**	− 0.82**	0.004	0.901**	0.606**	–				
7. Prospect/refuge	0.766	0.976**	− 0.904**	0.318**	0.695**	0.47**	0.898**	–			
8. Valence	0.792	0.955**	− 0.834**	0.01	0.894**	0.6**	0.994**	0.906**	–		
9. Magenta–green	0.482	− 0.431**	0.492**	− 0.261**	− 0.28**	− 0.216**	− 0.387**	− 0.461**	− 0.398**	–	
10. Blue–yellow	0.592	0.134**	− 0.299**	0.273**	− 0.11*	− 0.11*	0.038	0.206**	0.052	0.358**	–
11. Fractal dimension	0.491	− 0.027	− 0.025	− 0.076	− 0.08	− 0.221**	− 0.006	− 0.014	− 0.011	0.114*	− 0.179**

** Correlation is significant at the 0.01 level (two-tailed)

* Correlation is significant at the 0.05 level (two-tailed)

^a^ Split-half reliability conducted using the Spearman–Brown correction. The same method was applied to the calculated properties as to the rated properties (i.e., each subset contained images from all image subcategories). Notably, the calculated properties are less reliable than the rated properties, likely due to the variability in our images across these calculated properties

### Relationships between participant ratings and selected physical properties

An important factor in understanding how nature may be restorative is to understand the relationship between ratings for different properties, and whether these interact with key calculated properties. Therefore, correlations were conducted (using JASP) to further understand the relationship between the rated properties and the calculated physical properties of fractal dimensionality as well as the a* (green–magenta) and b* (blue–yellow) channels of the CIELAB parameters (see Table 2). We explored the relationship between the fractal dimensionality and colour properties of images with participant-rated properties due to the reported associations with cognitive restoration and environment preference. These were planned correlations, although no explicit predictions were made. Spearman’s correlations were used due to the non-normal distribution of the data; all values shown are uncorrected.

### Differences in ratings of properties between image sub-categories

In addition to the broad categories of nature and urban control, we had sub-categories of nature images. Each participant rated at least one image from each category, providing data for all rated properties. The average number of images seen by each participant for each subcategory is displayed in Table 3. To assess the internal consistency (reliability) of the ratings across the subcategories of images, we calculated Cronbach’s alpha reliability statistics using JASP for each rated property. Results showed good consistency in ratings between the image types (all *α* ≥ 0.853, see Table 3). To investigate whether there were broad differences in how the different image types were rated, we compared aggregate data (i.e., using the ratings for each image as an average across participants) for each image type using individual univariate ANOVAs with the rating categories as the dependent variable and image type as fixed factors. For all ANOVAs there was a significant effect of image category (all *F* values ≥ 35.715, all *p* values < . 001). Focusing on the differences between each nature type and the control images, we see that for pleasantness, there is no statistical difference in ratings between the urban environments (control) and caves (*p*_holm_ = 0.195). However, all other image types are rated significantly more pleasant than control images (all *p*_holm_ ≤ 0.002). For the approach/avoid ratings, participants reported significantly greater avoidance tendencies for caves than for all other environments, including the control environment (all *p*_holm_ < 0.001). Further, the desert environment was rated statistically equivalent to control (*p*_holm_ = 0.957), as was the snow environment (*p*_holm_ = 0.059); all other environments were considered more approachable than the control environment (all *p*_holm_ < 0.001). For valence, the control images were rated significantly lower than all image types except caves (caves vs control, *p*_holm_ = 0.435, all others, *p*_holm_ < 0.001). For arousal, caves were rated significantly more arousing than all other image types including control (all *p*_holm_ < 0.001). Control images were also rated as significantly more arousing than all other image types, except caves (all *p*_holm_ < 0.001). For familiarity, the control images were considered significantly more familiar than all other image types (all *p*_holm_ ≤ 0.018). For interest, the control images were not rated significantly different to deserts (*p*_holm_ = 0.057), but all other image types were rated significantly more interesting than control (all *p*_holm_ < 0.001). For mystery (asked in terms of there being more to discover), the control images were not rated significantly different to caves, fields, woodlands, managed landscapes, or snowy landscapes (all *p*_holm_ ≥ 0.135). Water, deserts, and mountains were considered significantly more mysterious than control images (*p*_holm_ < 0.001). Finally, in terms of prospect and refuge (rated in terms of feeling uneasy vs at ease in the environment), participants indicated that they would feel more uneasy in the cave than in all other environments, including the control environment (all *p*_holm_ < 0.001). Further, the desert environment was rated as somewhere the raters would feel more uneasy than the control (*p*_holm_ = 0.028), and the snow environment was rated equivalent to control (*p*_holm_ = 0.909); all other environments were rated as somewhere they would feel more at ease than the control environment (all *p*_holm_ < 0.001). Ratings of images by category are shown in Table 3.

**Table 3:** Average ratings^a^ of images by category

	Number of images/category (total)	Number of images rated/participant (*M*)^b^	Rating pleasantness (*M*, *SD*)	Rating Approach/Avoid (*M*, *SD*)	Rating Valence (*M*, *SD*)	Rating Arousal (*M*, *SD*)	Rating Familiarity (*M*, *SD*)	Rating Interest (*M*, *SD*)	Rating Mystery (*M*, *SD*)	Rating Prospect/Refuge (*M*, *SD*)
Control	65	13	5.08 (0.66)	5.26 (0.71)	5.14 (0.59)	5.23 (0.37)	6.10 (0.45)	4.99 (0.79)	5.70 (0.87)	5.21 (0.61)
Cave	17	3	4.71 (0.92)	4.32 (0.89)	4.92 (0.89)	5.77 (0.38)	3.03 (0.37)	5.75 (0.80)	6.14 (0.60)	3.76 (0.76)
Desert	31	6	5.60 (0.52)	5.17 (0.56)	5.71 (0.51)	4.56 (0.31)	3.42 (0.48)	5.39 (0.60)	5.01 (0.73)	4.80 (0.53)
Fields	28	6	7.06 (0.48)	6.94 (0.54)	7.06 (0.48)	2.97 (0.36)	5.73 (0.61)	6.11 (0.52)	5.33 (0.55)	6.98 (0.48)
Managed	70	14	6.57 (0.92)	6.54 (0.93)	6.62 (0.85)	3.47 (0.66)	5.37 (0.62)	5.93 (0.59)	5.47 (0.85)	6.53 (0.89)
Mountains	35	7	6.80 (0.58)	6.30 (0.70)	6.85 (0.52)	4.06 (0.58)	4.28 (0.52)	6.74 (0.53)	6.50 (0.50)	5.90 (0.72)
Snow	32	6	6.42 (0.47)	5.65 (0.65)	6.37 (0.44)	4.32 (0.52)	3.79 (0.78)	6.29 (0.45)	5.61 (0.50)	5.22 (0.69)
Water	108	22	7.39 (0.52)	7.09 (0.67)	7.37 (0.47)	3.39 (0.60)	4.98 (0.58)	7.14 (0.47)	6.57 (0.49)	6.83 (0.68)
Woodland	114	23	6.37 (0.59)	6.21 (0.58)	6.45 (0.53)	3.63 (0.43)	5.56 (0.53)	5.98 (0.54)	5.90 (0.47)	6.07 (0.56)
Reliability across categories (Cronbach’s α)	-	-	0.871	0.866	0.874	0.892	0.867	0.861	0.864	0.853

^a^ Note, images were rated on a scale of 1–9

^b^ All rounded to nearest whole number, for all, *SD* < 1

### Comparing physical properties between nature and control images

To determine whether there were differences in the calculated physical properties between the nature images and the urban control images in the database, we compared luminance, contrast, entropy, a* and b* channels of CIELAB parameters, and fractal dimension for the nature (*n* = 435) and urban control images (*n* = 65) using independent-samples *t*-tests. As we made no predictions, two-sided *p* values are used. For luminance, a*, b*, and fractal dimension, Levene’s test indicated unequal variance, so Welch’s test was used. Contrast and entropy were assessed with a Student *t*-test. Analysis was carried out in R (version 4.2.2) using the “t.test” function. Unless indicated, reported *p* values are uncorrected.

Nature images were significantly different from control images on the following indices: contrast (control: *M* = 0.240, *SD* = 0.037; nature: *M* = 0.229, *SD* = 0.042; *t*(498) = − 2.092, *p* = 0.037,* d* = − 0.279), with lower contrast in the nature images; the a* channel (green–magenta) of the CIELAB parameters (control: *M* = 1.168, *SD* = 2.379; nature: *M* = − 1.845, *SD* = 5.712; *t*(199.834) = − 7.483, *p* < 0.001, *d* = − 0.559), indicating that more green colour appeared in nature images; and the b* channel (yellow–blue) of the CIELAB parameters (control: *M* = 0.158, SD = 6.331; nature: *M* = 5.853, *SD* = 12.400; *t*(151.093) = 5.783, *p* < 0.001, *d* = 0.484), indicating that more yellow colour appeared in nature images.

No significant differences between control and nature images were found for luminance (control: *M* = 0.444, *SD* = 0.081; nature: *M* = 0.439, *SD* = 0.106; *t*(99.947) = − 0.389, *p* = 0.698, *d* = − 0.043), entropy (control: *M* = 7.537, *SD* = 0.252; nature: *M* = 7.516, *SD* = 0.276; *t*(498) = − 0.578, *p* = 0.563, *d* = − 0.077), or fractal dimension (control: *M* = 1.858, *SD* = 0.019; nature: *M* = 1.856, *SD* = 0.023; *t*(95.556) = − 0.972, *p* = 0.333, *d* = − 0.111).

Higher fractal dimensions in nature scenes have been associated with cognitive restoration via increased perceptual fluency (Joye et al., 2016), and changes in eye movements (Franěk et al., 2019). The lack of difference between fractal dimensions of nature and control images here therefore warrants further investigation. The result may be due to the variety of diverse categories of nature images included in the image set. As such, we performed a further one-way ANOVA to assess differences in fractal dimension of each category of images. A significant effect of category on fractal dimension was observed, *F*(8,491) = 21.86, *p* < 0.001, with Tukey-adjusted pairwise comparisons indicating differences between the control images (*M* = 1.858) and caves (*M* = 1.823; *p* < 0.001), deserts (*M* = 1.875; *p* = 0.006), snow (*M* = 1.876; *p* = 0.003), and woodland images (*M* = 1.843; *p* < 0.001); all other comparisons, *p*s > 0.09.

### Effects of participant characteristics

We compared individual participant ratings for valence, arousal, approach, and pleasantness for the images of nature (*n* = 435) in comparison to the urban control images (*n* = 65) in the database using paired-samples *t*-tests in JASP 0.15 (JASP Team, 2021). Results were in line with the aggregate data, confirming that participants rated the images of nature significantly differently to the urban images across all ratings. Participants rated the nature images as evoking higher positive valence (*M* = 6.656, *SD* = 1.024) than the urban control images (*M* = 5.137, *SD* = 1.363), *t*(800) = 26.117, *p* < 0.001, *d* = 0.923. Participants rated the nature images as evoking lower arousal (*M* = 3.738, *SD* = 1.193) than the urban control images (*M* = 5.230, *SD* = 1.324), *t*(800) = 26.567, *p* < 0.001, *d* = 0.939. Participants rated the nature images as evoking higher approach (*M* = 6.346, *SD* = 1.154) than the urban control images (*M* = 5.259, *SD* = 1.559), *t*(800) = 15.535, *p* < 0.001, *d* = 0.549. Finally, participants rated the nature images as evoking higher pleasantness (*M* = 6.621, *SD* = 1.060) than the urban control images (*M* = 5.079, *SD* = 1.384), *t*(800) = 26.097, *p* < 0.001, *d* = 0.922.

In addition, we compared individual participant ratings on the other factors between the nature images and the control images in our study. Again, ratings were in line with those seen for the aggregate data. Participants overall rated the nature images as evoking a significantly lower feeling of familiarity (*M* = 4.911, SD = 1.267) than the urban control images (*M* = 6.098, *SD* = 1.569), *t*(800) = 18.296, *p* < 0.001, *d* = 0.646. Participants rated the nature images as significantly more interesting (*M* = 6.301, *SD* = 1.155) than the urban control images (*M* = 4.987, *SD* = 1.597), *t*(800) = 19.824, *p* < 0.001, *d* = 0.700. Participants also rated the nature images as having significantly more to discover (higher mystery) (*M* = 5.932, *SD* = 1.197) than the urban control images (*M* = 5.695, *SD* = 1.537), *t*(800) = 3.524, *p* < 0.001, *d* = 0.125. Finally, we found that the participants overall rated the nature images as being of somewhere they were more likely to feel at ease (prospect/refuge) (*M* = 6.135, *SD* = 1.171) compared to the urban control images (*M* = 5.203, *SD* = 1.529), *t*(800) = 13.225, *p* < 0.001, *d* = 0.467.

To determine whether there were any effects of the participant characteristics on ratings, we looked at the rating of pleasantness for control and nature images. We chose to focus on pleasantness, as this is a basic indicator of whether someone liked the environment type. As such, we created a score for pleasantness by taking the rating for the control images from the nature images; thus a higher number would indicate a greater preference for nature.

Due to the large number of measures, and no explicit predictions or expectations around interactions, we ran simple ANOVAs to test the different fixed demographic factors. Gender identity did not significantly affect ratings, *F*(2, 798) = 1.859, *p* = 0.156, *ηp*^2^ = 0.005. Neither did education, *F*(6, 794) = 0.923, *p* = 0.478, *ηp*^2^ = 0.007. Interestingly, there was a significant effect related to geographical region (in terms of Prolific groupings, see Table 4), *F*(7, 793) = 10.940, *p* < 0.001, *ηp*^2^ = 0.088. Those from the African nations showed the smallest difference between images of nature and control images on pleasantness, with the difference between ratings being significantly lower than all other regions (all *p*_holm_ ≤ 0.007). The largest difference came from the UK participants, though this was only significantly larger than the African, Asian (excluding Australia), and South/Central American samples (*p*_holm_ ≤ 0.010). Importantly, using paired-samples* t*-tests to compare, all regions showed a significant difference in ratings between the nature and control images (all *p*s ≤ 0.003).

**Table 4:** Ratings of images by region for pleasantness

Regions	Control (*M*, *SD*)	Nature (*M*, *SD*)
Africa	5.76 (1.55)	6.25 (1.17)
Arab States and Middle East	5.09 (1.34)	6.85 (1.11)
Asia and South Pacific	5.16 (1.33)	6.40 (1.16))
Australia and New Zealand	4.80 (1.34)	6.65 (1.11)
Europe	5.01 (1.27)	6.85 (0.91)
North America	4.86 (1.50)	6.62 (1.06)
South and Central America	5.27 (1.23)	6.70 (1.00)
UK	4.53 (1.23)	6.76 (0.88)

Finally, we ran a backwards stepwise regression to test whether the difference in pleasantness rating between nature and control environments could be predicted by participant age, perceived socio-economic status (as measured by the MacArthur scale), and scores on the urban versus rural scale in terms of where participants lived, their current location, where they spent most of their time, and whether they liked spending leisure time in rural and urban environments. The regression showed that age, preference for spending time in urban environments, preference for spending time in rural environments, and current location significantly predicted pleasantness ratings for urban versus nature environments, *F*(4, 796) = 19.364, *p* < 0.001, *R*^2^ = 0.089, using a regression equation: pleasantness rating = 1.923 + (0.065 × leisure rural) + (− 0.118 × leisure urban) + (− 0.071 × current location) + (0.016 × age).

## Discussion

The current study is an account of the development of the SNED comprising 500 images of natural and urban environments rated for mystery, interest, feeling at ease, familiarity, pleasantness, valence, arousal, and approach–avoidance, with additional information detailing the physical properties of the images. The images represent six broad categories of natural environments: water, woodlands, landscapes, caves, managed landscapes (i.e., farmland, reservoirs, and parks), and control (i.e., urban). The landscapes category can be further split into four subcategories: desert, mountain, field, and snow. This approach allowed us to capture a range of natural scenes.

We developed the SNED to provide a standardized database of natural environment images which considered the physical properties of an environment deemed important in cognitive restoration research. To allow consideration of inter-individual and cultural influences on image ratings, we provide demographic information including age, gender identity, ethnicity, geographical location, education background, economic status, and general environment preference for each of our participants. Participants also provided information about the environment where they lived and spent most of their leisure time. The SNED is therefore unique in that it represents an extension beyond the limits of other available pictorial databases (e.g., e-NatPOEM; Dal Fabbro et al., 2021), offering a more detailed description of both the image properties and characteristics of the individuals providing the image ratings.

The affective space of the ratings for nature images was in line with that seen in e-NatPOEM, with the nature images generally evoking higher positive valence and lower arousal ratings than control (urban) images. Further, the nature images were rated significantly higher in approach tendencies and pleasantness than control images. Participants also rated the nature images as evoking lower feelings of familiarity but being more interesting than the control images. Finally, nature images were rated as having more to discover (i.e., higher mystery), and participants indicated that they would feel more at ease (i.e., prospect/refuge) in the nature environments when compared with the control images. Considered together, these results evidence differences in perceived restorative properties between nature and control (urban) images, offering validation for the SNED. Importantly, we also observed good reliability across our rated properties.

To provide further validation, we conducted a visual comparison of our ratings with similar images from the NAPS (Marchewka et al., 2014) and e-NatPOEM (Dal Fabbro et al., 2021) in relation to affective space. Like the NAPS and e-NatPOEM, our database shows a linear relationship between valence and arousal. Further, as is the case in the e-NatPOEM (Dal Fabbro et al., 2021), our images of nature fall largely in the upper left quartile of affective space, with low arousal and high valence ratings. Our control images lie more centrally than those of the e-NatPOEM, which show high arousal and low valence. This may be due to the types of images used as controls in e-NatPOEM, which include images containing destruction and animals (as well as urban environments).

In terms of effects of participant characteristics, while we saw some minor differences in terms of magnitude of ratings, the general preference for nature over urban environments appears consistent across the populations. Interestingly, ratings of pleasantness did seem to be moderated by factors related to age (older people showed a stronger preference for nature) and, as would be expected, preferences around urban versus rural environments. Researchers focusing on variation in individual characteristics as an avenue for further investigation may therefore find our database particularly valuable.

The SNED is intended primarily for use in research, and analysis of the ratings presented here are intended to offer insight into how the database can facilitate rigorous and robust scientific enquiry based on key theoretical perspectives within this research area. Crucially, our results showed that image ratings across the different factors were highly correlated; therefore, as may be expected, each of the factors that are considered important by theoretical restoration research are highly related to one another. Interestingly, the only rated properties that did not correlate with each other were mystery, defined here as there being a lot to discover in the environment, and familiarity. This indicates that the properties put forward by theoretical restoration research may be capturing the same underlying features of an environment. For example, the properties of mystery and interest both capture the characteristic of fascination (Hartig et al., 1997), and it is arguable that this also relates to general pleasantness of an environment, as well as how at ease we may feel within it. In addition, a feeling of familiarity has been associated with how much we like something (Zajonc, 1980), as well as how at ease we may feel. Ratings of valence, arousal, and approach–avoidance are also known to be highly corelated (Campbell et al., 2021), and ratings on these factors will be influenced by properties related to the restorative factors (i.e., mystery, familiarity, interest, feeling at ease) and pleasantness. However, while we show an overall correlation across these properties, individual images will possess different levels of these properties, and it is important to understand why and how these differences affect the restorative properties of the images and the environments they represent. We believe such an endeavour will be greatly facilitated by the availability of the SNED.

Indeed, while overall our nature environments were preferred to the urban environments, we found that cave environments were not particularly well liked when compared with other nature images and the control images. One explanation could be that caves do not offer the comparative soft fascination as seen in other environments that can restore attentional capacities according to attention restoration theory (Kaplan, 1995). Alternatively, it is possible that the cave images presented in the SNED were not good representations of what a typical cave environment encompasses in terms of giving a sense of prospect and refuge which would lead to a reduction in stress as proposed in stress reduction theory (Ulrich, 1983; Ulrich et al., 1991). Water images stood out in most categories as achieving the most favourable ratings, including in terms of pleasantness, valence, and approach, which is in line with research that has shown that blue spaces are among the most restorative contexts (e.g., White et al., 2020). However, fields were rated most relaxing, potentially due to their higher perceived familiarity (highest for the nature images). Collectively, our findings suggest that there are distinct differences within the perceived ratings of nature images, providing useful avenues for future research exploring natural environments and cognitive restoration.

Higher fractal dimensions in nature scenes have previously been associated with cognitive restoration via increased perceptual fluency (Joye et al., 2016) and changes in eye movements (Franěk et al., 2019). We therefore compared our nature and control images based on their fractal dimension. We found that there was no overall difference between nature images and our urban images related to fractal dimensions. However, due to the variability in image types in the database, we broke the images down into the six broad categories covered. We found that there were no differences between control images and images containing water, managed landscapes, mountains, and fields. Our desert and snow images had significantly greater levels of fractal dimension than our control images, whilst in contrast, caves and woodland environments had significantly less fractal dimension than our control images. Desert and snow images may contain a greater number of repeating patterns that, crucially, occur at different scales (e.g., dunes or drifts, and ripples in sand/snow) and will therefore register a higher fractal dimension. It is worth noting, however, that the variation in fractal dimension of images as estimated in this database is low. Our findings suggest that if fractal dimensionality within nature scenes is related to perceptual fluency and cognitive restoration, this may only be for specific nature environments (e.g., deserts and snow). Further, some natural environments appear to have reduced fractal dimension (compared to control urban images), and therefore any associated benefits of these environments on wellbeing are less likely to be driven by fractal dimension. Of our rated properties, only mystery was correlated with fractal dimension, where increased fractal dimension was associated with increased mystery ratings. Further investigation specifically manipulating both fractal dimension (within natural scenes) and mystery is needed to more thoroughly test the predictions made by perceptual fluency theory, with this database facilitating such research.

Researchers who investigate the effect of image properties on their restorative effects often use image manipulations to alter aspects such as spatial frequency (e.g., Menzel & Reese, 2021; Valtchanov & Ellard, 2015). Whilst such studies offer valuable insight, they rely on heavily manipulated images that may fail to accurately reflect the real-world environment. The SNED contains 500 images that have information on their luminance, contrast, entropy, CIELAB colour space parameter values, and fractal dimensionality and are rated on aesthetic properties, allowing for a controlled manipulation and assessment of the effect of variation in properties without manipulating the actual image. It is anticipated that future researchers will exploit the SNED as a means to test and analyse the effect of these variations in relation to particular image properties.

At the core of the database’s strengths is the range and diversity of the images included. This was achieved using the online image sharing platform Flickr, so that all images are in the public domain and are therefore free for use by researchers. This means that we lack comprehensive information regarding camera settings used to capture the images or whether the images were edited by the photographers prior to uploading. Whilst screening was conducted for obvious indicators of editing, it remains possible that images were edited to improve or change their aesthetic appearance. Further, while many of the images have EXIF information available with the original image, this information is not available for all images, so it is important to remain conscious of these potential limitations when using the database.

Research suggests that real nature and simulated nature show no significant difference in their restorative effect (Kjellgren & Buhrkall, 2010), with images affording a greater degree of control over confounding variables not achievable in real nature environments. However, criticism exists in relation to the use of images in restoration research, as these only address the visual effects of nature, when other sensory stimuli, particularly aural, have also been shown to have a restorative effect both alone and when combined with images of nature (Deng et al., 2020; Ratcliffe, 2021). It may therefore be important in future research to combine image databases such as the SNED with soundscape databases of natural sounds to achieve greater ecological validity, as in real nature, both visual and aural information is available.

The SNED presented here is intended to support researchers in their investigations of the restorative effects of nature on cognition and health. Comprising 500 high-quality, standardized photographs capturing a variety of possible natural environments across the seasons, with data on participant ratings and image properties, the SNED exists as the most comprehensive nature image database currently available. Normative scores for user-rated characteristics of fascination, refuge and prospect, compatibility, preference, valence, arousal, and approach–avoidance, as well as data on physical properties of the images, specifically luminance, contrast, entropy, CIELAB colour space parameter values, and fractal dimensions, will enable researchers to better test posited theories explaining the restorative effects of nature, enabling a more comprehensive understanding of how and when natural environments facilitate restoration. With the potential for such advances to lead to the development of more effective nature-based therapies and social prescribing, the SNED is offered as open access. We therefore encourage researchers to use the images and associated properties presented to facilitate their own research seeking a better understanding of how we can harness the benefits to improve functioning, health, and wellbeing.

## Data Availability

All data and materials are available at the project’s Open Science Framework page (https://osf.io/qm42t/) and in the project GitHub (https://github.com/Salford-PsyTech/Salford_Nature_Environments_Database).

## Footnotes

1 Recently, a database of soothing pictures has also been created (MacLennan et al., 2023), which contains pictures from natural scenes, including water features, flowers and trees, landscapes, animals, sky, other images, people, buildings, and snow. However, these images were not chosen based on any environmental factors or in relation to key theoretical perspectives from restoration research, again showing the need for the work presented here.

## Code availability

All code is available at the project’s Open Science Framework page (https://osf.io/qm42t/).

## Declarations

### Open practices statement

The data and materials for all experiments are available at the projects Open Science Framework page (https://osf.io/qm42t/) and in the project GitHub (https://github.com/Salford-PsyTech/Salford_Nature_Environments_Database). The research was preregistered (https://osf.io/sbqg8).
